# Cathepsin L promotes angiogenesis by regulating the CDP/Cux/VEGF-D pathway in human gastric cancer

**DOI:** 10.1007/s10120-020-01080-6

**Published:** 2020-05-09

**Authors:** Tao Pan, Zhijian Jin, Zhenjia Yu, Xiongyan Wu, Xinyu Chang, Zhiyuan Fan, Fangyuan Li, Xiaofeng Wang, Zhen Li, Quan Zhou, Jianfang Li, Bingya Liu, Liping Su

**Affiliations:** 1 Department of Surgery, Shanghai Key Laboratory of Gastric Neoplasms, Shanghai Institute of Digestive Surgery, Ruijin Hospital, Shanghai Jiao Tong University School of Medicine, Shanghai, China; 2 Department of General Surgery, First People’s Hospital, Shanghai General Hospital, Shanghai Jiao Tong University School of Medicine, Shanghai, China

**Keywords:** CTSL, Cux, VEGF-D, Angiogenesis

## Abstract

**Background:**

Increasing evidence indicates that angiogenesis plays an important role in tumor progression. The function of cathepsin L (CTSL), an endosomal proteolytic enzyme, in promoting tumor metastasis is well recognized. The mechanisms by which CTSL has promoted the angiogenesis of gastric cancer (GC), however, remains unclear.

**Methods:**

The nuclear expression levels of CTSL were assessed in GC samples. The effects of CTSL on GC angiogenesis were determined by endothelial tube formation analysis, HUVEC migration assay, and chick embryo chorioallantoic membrane (CAM) assay. The involvement of the CDP/Cux/VEGF-D pathway was analyzed by angiogenesis antibody array, Western blot, co-immunoprecipitation (Co-IP) and dual-luciferase reporter assay.

**Results:**

In this study, we found that the nuclear CTSL expression level in GC was significantly higher than that in adjacent nontumor gastric tissues and was a potential important clinical prognostic factor. Loss- and gain-of-function assays indicated that CTSL promotes the tubular formation and migration of HUVEC cells in vitro. The CAM assay also showed that CTSL promotes angiogenesis of GC in vivo. Mechanistic analysis demonstrated that CTSL can proteolytically process CDP/Cux and produce the physiologically relevant p110 isoform, which stably binds to VEGF-D and promotes the transcription of VEGF-D, thus contributing to the angiogenesis of GC.

**Conclusion:**

The findings of the present study suggested that CTSL plays a constructive role in the regulation of angiogenesis in human GC and could be a potential therapeutic target for GC.

**Electronic supplementary material:**

The online version of this article (10.1007/s10120-020-01080-6) contains supplementary material, which is available to authorized users.

## Background

Gastric cancer (GC) is one of the most common and deadliest types of cancer worldwide, with particularly high morbidity and mortality in China [3, 7]. Nearly, half of all patients with GC develop metastases. At present, surgery is the major curative treatment for GC; however, the prognosis of GC remains poor [2, 35]. Therefore, the exploration of new GC treatment strategies is needed.

Cathepsins, containing about 15 classes in human, are defined as protease enzymes with versatile functions [24]. These enzymes are vital for normal physiological processes, such as innate immunity, apoptosis, autophagy, and angiogenesis [4]. Dysregulated cathepsins can also contribute to numerous pathologies, including arthritis, pancreatitis, atherosclerosis, and cancers [6, 24]. In GC, most of cathepsins have reported to be overexpressed and are associated intimately with the proliferation and metastasis of GC cells, lymph node metastasis, and poor prognosis [13, 14, 19]. Cathepsin L (CTSL), one of the human cathepsin proteases, is primarily present within the lysosomes or secretory vesicles and predominantly exhibits endoproteolytic activity [5, 9, 31]. Our study, however, showed that CTSL also is found in the nucleus of GC; overexpression of nuclear CTSL in tumor tissues was significantly associated with differentiation, local invasion, TNM stage, lymph metastasis, and shorter survival of GC patients. Moreover, loss- and gain-of-function assays demonstrated that CTSL promotes tumor angiogenesis in GC.

Accumulating evidence has revealed that tumor progression is strongly associated with angiogenesis, which plays a critical role in tumor growth, relapse, and metastasis. Vascular endothelial growth factor (VEGF) is the key regulator of angiogenesis, and tumor-derived VEGFs play a central role in tumor angiogenesis [16, 27]. VEGF-D, a member of the VEGF family, is identified as a lymph-angiogenic growth factor and participates in the growth of lymphatic vessels as well as in angiogenesis [1, 28]. VEGF-D binds to the tyrosine kinase receptors (TKRs) VEGFR-2 and VEGFR-3 that are expressed specifically on the surface of GC cells, which then activates the downstream signaling pathways, including the phosphatidylinositol 3 kinase (PI3K)/AKT, protein kinase C (PKC), and mitogen-activated protein kinase (MAPK) pathways [21], to induce angiogenesis within the solid tumors, promoting tumor growth and distant metastasis [17, 34]. In our study, we found that CTSL could proteolytically cleave the CDP/Cux p200 and generate CDP/Cux p110, which contributed to the transcriptional activation of VEGF-D and promoted tumor angiogenesis in GC. These results indicated the novel mechanism of CTSL in the progression of GC and revealed that it could act as a new therapeutic target in cancer therapy.

## Methods

### Cell culture and patient samples

The GC cell lines AGS and NCI-N87 used in our study were purchased from the American Type Culture Collection (ATCC) and SUN-1, MGC803, MKN45, MKN28, HGC27, and GES-1 were purchased from Shanghai Institutes for Biological Sciences, Chinese Academy of Sciences. All GC cell lines were authenticated by short-tandem repeat analysis and had negative results for mycoplasma. SUN-1, AGS, NCI-N87, MKN45, MKN28, and HGC27 were cultured at 37 °C in a humidified atmosphere of 5% CO_2_ with RPMI-1640 medium containing 10% fetal bovine serum with 100 U/ml penicillin and 100 U/ml streptomycin. GES-1 and MGC803 were cultured at 37 °C in a humidified atmosphere of 5% CO_2_ with Dulbecco’s Modified Eagle Medium (DMEM) containing 10% fetal bovine serum with 100-U/ml penicillin and 100-U/ml streptomycin.

A total of 174 patients in this study underwent resection of the GC at Shanghai Ruijin Hospital. All samples were obtained with the patients’ informed consent, and the samples were histologically confirmed.

### RNA extraction and qRT-PCR

Total RNA was extracted with Trizol reagent (Invitrogen, Carlsbad, CA, USA), which was reversely transcribed into cDNA using a Reverse Transcription system (TOYOBO, Kita-ku, Osaka, Japan). Quantitative real-time polymerase chain reaction (qRT-PCR) was performed to quantify CTSL and VEGF-D mRNA levels with the SYBER Green PCR Master Mix (Applied Biosystems, Foster City, CA, USA). Glyceraldehyde 3-phosphate dehydrogenase (GAPDH) were used as the endogenous reference and used the ${2}^{{ - \left( {\Delta \Delta C_{{\text{t}}} } \right)}}$ method to calculate the relative abundance of RNA compared with GAPDH expression.

### Western blot

Protein was extracted from cells using radioimmunoprecipitation (RIPA) buffer (Thermo, Rockford, IL, USA) and cell debris were removed by centrifugation. The extracts were quantified using Pierce BCA Protein Assay Kit (Thermo) and then boiled the extracts in sodium dodecyl sulfate (SDS) gel-loading buffer containing 10% β-mercaptoethanol. The proteins were separated in 10% SDS–polyacrylamide gel electrophoresis (SDS–PAGE) gels and transferred them onto immobilon polyvinylidene fluoride (PVDF) membranes (Millipore, Burlington, MA, USA), which were probed with rabbit mAbs against mouse GAPDH (1:5000; 60004-1-Ig; ProteinTech Group, Chicago, IL, USA), CDP/Cux1 (1:1000; sc514008X; Santa Cruz Biotechnology, Dallas, TX, USA), VEGF-D (1:1000; 26915-1-AP; ProteinTech Group, Chicago, IL, USA), and CTSL (1:1000, ab58991; Abcam, Cambridge, MA, USA) and then incubated with horseradish peroxidase (HRP)-conjugated goat anti-rabbit (SA00001-2) and anti-rabbit immunoglobulin G (IgG, SA00001-1) antibody (1:10,000; 13099-1-AP; ProteinTech Group, Chicago, IL, USA), which was followed by Pierce™ ECL Western Blotting Substrate (Thermo).

### Plasmids construction and transfection

Two CTSL small hairpin RNAs (shRNA, sh-CTSL #1 and sh-CTSL #2) and negative control (Ctrl) sequences were designed and constructed into a hU6-MCS-Ubiquitin-EGFP-IRES-puromycin plasmid. HGC27, MGC803, MKN28, and MKN45 cells were transfected with shRNA plasmids using Lipofectamine 3000 reagent (Invitrogen) according to the manufacturer’s protocol. CTSL cDNA was subcloned into the CN550-pLOV-EF1a-PuroR-CMV-eGFP-2A-3FLAG plasmid (Obio Technology Co. Ltd., Shanghai, China). CTSL plasmid and corresponding empty vector were transfected the into GC cells (HGC27, MGC803, AGS, and SUN-1) using Lipofectamine 3000 reagent (Invitrogen) following the manufacturer’s protocol. The stably transfected cell lines were selected by puromycin.

### Immunohistochemistry staining

IHC staining was performed on the tissue microarray according to a previously reported standard protocol used in Shanghai Institute of Digestive Surgery [36]. In brief, freshly cut 5-μm-thick tissue microarray was stained with antibodies against CTSL (1:200, ab203028, Abcam, Cambridge, MA, USA), VEGF-D (1:1000; 26915-1-AP; ProteinTech Group, Chicago, IL, USA) and PECAM1 (1:200, ab268100; Abcam, Cambridge, MA, USA). Two independent board-certified pathologists evaluated the protein expression levels in an unbiased fashion. The staining intensity and percentage were used to score the overall tissue sections and the intensity was graded as follows: 0 points (no staining), 1 point (light-brown staining), 2 points (brown staining), and 3 points (dark-brown staining). We divided the percentage of positive cell number into four grades: 1 points (< 5%), 2 point (5–30%), 3 points (31–60%), and 4 points (61–100%). We calculated the staining score as follows: overall staining score = intensity score × percentage score. A final score ≤ 3 was considered to be negative staining, and a score > 3 was considered to be positive staining.

### Transient transfection and dual-luciferase reporter assay

HGC27-Vector and HGC27-p110Cux1 cells were seeded in 24-well plates and transfected the cells with pGL3-VEGF-D, pGL3-VEGF-D-del 1, pGL3-VEGF-D-del 2, pGL3-VEGF-D-del 3, and pRL-TK plasmid using a Lipofectamine™ 3000 Transfection Reagent (Invitrogen) according to the manufacturer’s instructions. Three days after transfection, dual-luciferase reporter assay was performed using Dual-Luciferase^®^ Reporter Assay System (Promega, Madison, WI, USA) according to the manufacturer's instructions.

### Endothelial tube formation analysis

Human umbilical vein endothelial cells (HUVECs) were cultured in tumor supernatants from each cell line in 96‐well plates at a density of 1 × 10^4^ cells per well for 6 h. Plates were pre-coated with 50-μL Matrigel (R&D Systems, 3533-010-02) at 37 °C for 1 h. After a 6-h incubation, tubule images were acquired and analyzed by Image‐Pro Plus software, which we quantified by counting the number of cell junctions and tubes in 10 randomly chosen fields of view. Data were obtained from three independent experiments.

### HUVEC migration assay

HUVECs were suspended in serum-free medium (1 × 10^5^ cells/insert) and added to the upper chamber of the 24-well insert (membrane pore size, 8 μm; Corning Life Science, Tewksbury, MA, USA). Medium containing 10% serum was added to the lower chamber. After incubation for 12 h, the cells that had migrated to the bottom of the membranes were fixed and stained them with 0.1% crystal violet for 30 min.

### Chick embryo chorioallantoic membrane assay

A chorioallantoic membrane (CAM) was performed as previously described [8]. After cutting a round window into an egg, 30 μL of cell culture supernatant was dropped onto a filter paper disk and sealed with transparent tape for 3 days. On day 10, the eggs were photographed with a MacroPATH dissecting microscope (Milestone, Sorisole, Italy) and the number of blood vessels around the filter paper disc was counted.

### Angiogenesis antibody array

A Human Angiogenesis Antibody Array C1000 (RayBiotech, Norcross, GA, USA) was used to analyze the conditioned medium (CM), following the manufacturer's protocol. The results of the antibody array were quantified with a chemiluminescence system (Bio-Rad, Hercules, CA, USA) similar to the Western blotting procedure.

### In vivo tumorigenesis

Four-week-old male BALB/c nude mice were purchased from the Institute of Zoology Chinese Academy of Sciences and housed at a specific pathogen-free environment in the Animal Laboratory Unit, Ruijin Hospital, China. HGC27 cells transduced by sh-CTSL or sh-Ctrl (2 × 10^7^) were harvested and resuspended in 500 μL of PBS. Cells (4 × 10^6^/100 μL PBS) were subcutaneously or peritoneally injected into the mice. For tumorigenesis in vivo, all mice were killed after 28 days, and subcutaneous lumps were removed, imaged, and placed in 4% formaldehyde for paraffin embedding. For peritoneal dissemination model, all mice were killed after 30 days and peritoneal metastasis nodules were counted.

### Statistical analysis

All experiments were repeated at least three times, and the results were summarized as means ± SD. Student *t* test and one-way analysis of variance (ANOVA) were used to analyze the data and Chi-square tests were used to analyze categorical variables. All statistical tests were performed with SPSS 25.0 (SPSS, Chicago, IL, USA) and *p* values < 0.05 were regarded as indicating statistically significant differences (*p < 0.05, **p < 0.01, ***p < 0.001).

## Results

### Overexpressed nuclear CTSL is positively associated with vessel density in human GC tissues and predicts poor prognosis of GC patients

**Fig. 1:**
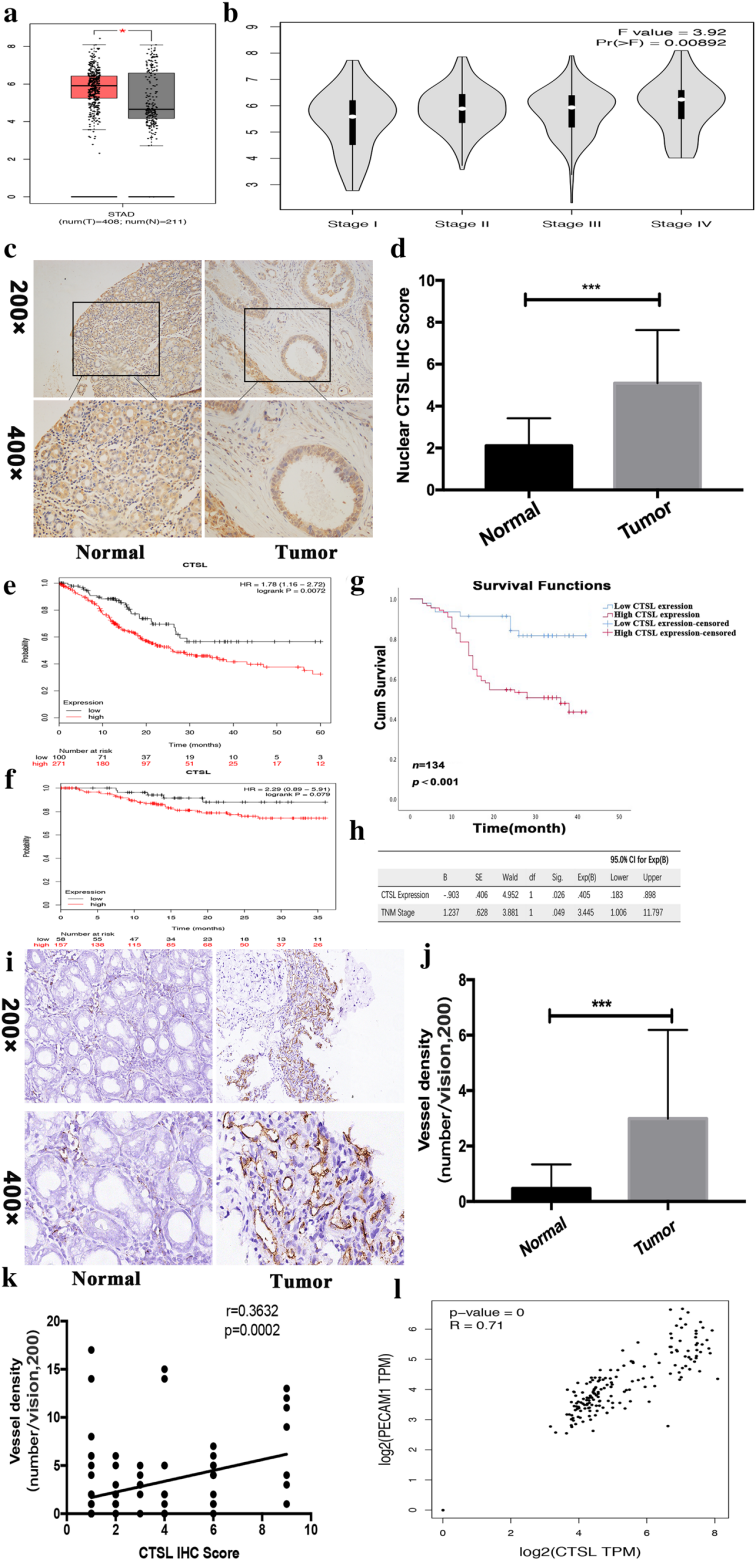
Overexpressed nuclear CTSL was positively associated with VD in human GC tissues and predicts poor prognosis of GC patients. **a** Box plots of 408 GC samples (red) and 211 normal samples (gray) revealed upregulation of CTSL in GC compared with normal tissue. **b** Violin plots revealed that CTSL expression was significantly associated TNM stages. **c** Representative IHC staining with CTSL antibody in GC and paired adjacent nontumor tissues. Magnification: × 200 and × 400. **d** Analysis of CTSL expression in 134 pairs of GC patients (GC cohort 1) showed that CTSL expression was significantly high in GC tissues compared with adjacent nontumor tissues. **e** The overall survival and **f** disease-free survival analyses were plotted using Kaplan–Meier Plotter for patients with GC. **g** Kaplan–Meier analysis of the overall survival of 134 patients with GC in GC cohort 1. The log-rank test revealed statistical significance between the low nuclear CTSL expression group (*n* = 46) and the high nuclear CTSL expression group (*n* = 88). **h** Multivariate analysis of different prognostic parameters for GC patients (GC cohort 1). **i** Representative IHC staining with PECAM1 antibody in GC and paired adjacent nontumor tissues. Magnification: × 200 and × 400. **j** Analysis of VD in 134 pairs of GC patients (GC cohort 1) showed that VD was significantly high in GC tissues compared with adjacent nontumor tissues. **k** Correlation analysis of CTSL and VD showed that CTSL was positively correlated with VD in GC. **l** Validation of the positive correlation between CTSL and VD in GEPIA

Previous studies have demonstrated that CTSL is involved in a wide range of human malignancies, including ovarian, breast, prostate, lung, pancreatic, and colon cancers [30]. In accordance with previous studies, bioinformatic analysis of CTSL expression profiles through Gene Expression Profiling Interactive Analyses (GEPIA; https://gepia.cancer-pku.cn) showed that the CTSL mRNA expression levels were significantly increased in majority of tumor types (Supplementary Fig. 1). Notably, CTSL was obviously upregulated in GC tissues (Fig. 1a). Moreover, bioinformatic analyses showed that expression of CTSL was positively related to TNM stage (Fig. 1b). We then examined CTSL protein expression in 134 pairs of GC tissues (GC cohort 1) and examined corresponding nontumor tissues by IHC. Typical immunostaining of CTSL in GC tissues and corresponding non-tumor tissues is shown in Fig. 1c. We primarily identified positive staining of CTSL protein in the cytoplasm and nuclei of GC cells. CTSL has been demonstrated to promote the development of GC [20, 22]; however, the exact role of nuclear CTSL in GC progression remains undefined. To further evaluate the function of nuclear CTSL in GC, we next analyzed the expression level of the nuclear fractions of CTSL in GC, and the results showed that the average expression score for the nuclear fractions of CTSL was significantly higher in GC tissues than that in adjacent nontumor tissues (Fig. 1d). Furthermore, we analyzed the correlation of nuclear CTSL with the clinical parameters of GC, as shown in Table 1. A high nuclear CTSL staining score was associated with more poor differentiation, local invasion*,* positive lymphatic metastasis status, and higher TNM stage. In addition, we assessed the correlation between CTSL expression and the survival of GC patients. Kaplan–Meier Plotter analysis (https://kmplot.com/analysis) showed that CTSL expression was inversely proportional to survival (Fig. 1e, f). We further identified that prognosis of cases with high nuclear CTSL expression was worse (Fig. 1g) and multivariate analyses of different prognostic parameters revealed that nuclear CTSL expression was an independent factor for overall survival (*p* = 0.026) (Fig. 1h). It has been reported that cysteine cathepsins play an important role in angiogenesis [25]. To further define the clinic role of CTSL in angiogenesis of GC, we detected the VD of GC tissues by IHC staining of PECAM1 (also called CD31, a marker of angiogenesis). The result showed that VD in tumor tissue was significantly higher than in non-tumor tissues (Fig. 1i, j), and further analysis showed that the nuclear CTSL expression was positively related to the VD of GC tissues (Fig. 1k). Similarly, bioinformatic analyses of the correlation between CTSL and PECAM1 through GEPIA (Fig. 1l) confirmed these findings, which indicated that nuclear CTSL may play a vital role in the angiogenesis of GC. Overall, these data suggest that upregulation of nuclear CTSL is positively correlated with VD in human GC and predicts poor prognosis of GC patients.

**Table 1:** Correlation between the clinicopathological features and expression of CTSL

Clinicopathologic parameters	Number of cases	Nuclear CTSL immunostaining		*p*
	134	Weak positive (*n* = 46)	Strong positive (*n* = 88)	
Gender				
Male	95	32	63	0.806
Female	39	14	25	
Age (years)				
≥ 60	75	30	45	0.119
< 60	59	16	43	
Tumor size (cm)				
≥ 5	95	32	63	0.806
< 5	39	14	25	
Lauren classification				
Intestinal	78	28	50	0.652
Diffuse	46	18	38	
Differentiation				
Poorly undifferentiated	23	17	6	< 0.001
Well moderately	111	29	82	
Local invasion				
T1, T2	20	16	4	< 0.001
T3, T4	114	30	84	
Lymph metastasis				
No	26	15	11	0.005
Yes	108	31	77	
TNM stage				
I, II	27	22	5	< 0.001
III, IV	107	24	83	

### CTSL promotes the tubular formation and migration of HUVEC cells

**Fig. 2:**
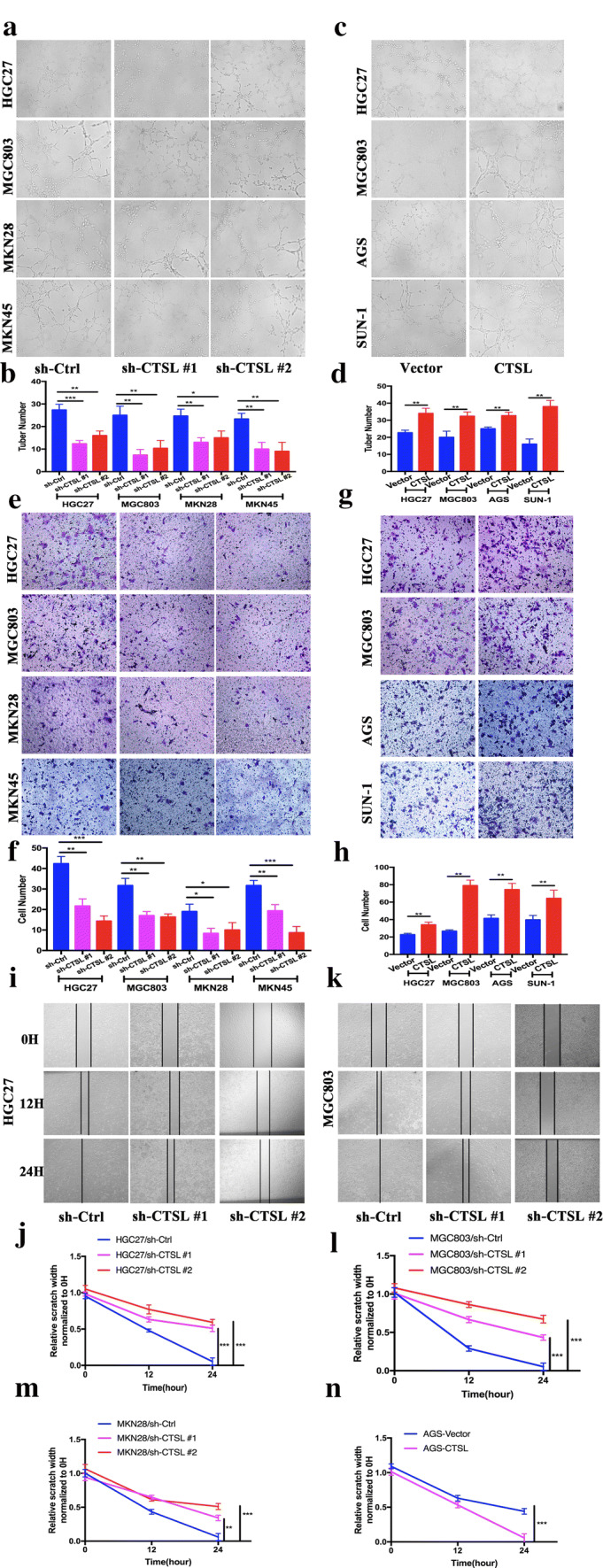
CTSL promoted the tubular formation and migration of HUVEC cells. **a**, **b** In vitro tubular formation to investigate the effect of CM from GC cells after transfection with CTSL shRNA plasmids. (MGC803, HGC27, MKN28, and MKN45) on HUVEC tubule formation. Data are presented as the mean ± SD; *n* = 3 independent experiments. **c**, **d** In vitro tubular formation to investigate the effect of CM from GC cells after transfection with CTSL-expressing plasmids (MGC803, HGC27, AGS, and SUN-1) on HUVEC tubule formation. Data are presented as the mean ± SD; *n* = 3 independent experiments. **e**, **f** In vitro Transwell migration assay to investigate the effect of CM from GC cells after transfection with CTSL shRNA plasmids (MGC803, HGC27, MKN28, and MKN45) on the metastatic abilities of HUVEC. Data are presented as the mean ± SD; *n* = 3 independent experiments. **g**, **h** In vitro Transwell migration assay to investigate the effect of CM from GC cells after transfection with CTSL-expressing plasmids (MGC803, HGC27, AGS, and SUN-1) on the metastatic abilities of HUVEC. Data are presented as the mean ± SD; *n* = 3 independent experiments. **i–m** Scratch wound-healing motility assays were performed to observe the changes in the migration of HUVEC after stimulation with CM from GC cells after transfection with CTSL shRNA plasmids (MGC803, HGC27, and MKN28) Data are presented as the mean ± SD; *n* = 3 independent experiments. **n** Scratch wound-healing motility assays were performed to observe the changes in the migration of HUVEC after stimulated with CM from GC cells after transfection with CTSL-expressing plasmids (AGS). Data are presented as the mean ± SD; *n* = 3 independent experiments

Given a potential link between CTSL overexpression and angiogenesis in GC, we determined whether knockdown or ectopic overexpression of CTSL in GC cells could affect the tubular formation and migration of endothelial cells. We identified the expression levels of CTSL in seven GC cell lines and one human gastric epithelial cell line (GES-1). Most GC cell lines expressed relatively high levels of CTSL (Supplementary Fig. 2A and Supplementary Fig. 2B). To determine whether the expression level of CTSL changed in GC cells and contributed to endothelial cells tubule formation and migration, we established an in vitro co-culture system (Supplementary Fig. 2C), in which HUVECs were indirectly cocultured with GC cells and separated from GC cells by a semipermeable membrane (pore size of 0.6 μm). Interestingly, we found that CTSL-knockdown GC cells elicited significantly less tubule formation by HUVECs compared with their respective control group (Fig. 2a, b); whereas, CTSL-overexpressing GC cells stimulated more tubule formation by HUVECs in comparison with their respective control group (Fig. 2c, d). Tumor cells may regulate HUVEC tubule formation by altering endothelial cell migration. We then performed Transwell assays, and the result revealed that overexpression of CTSL in GC cells promoted the migration of HUVECs (Fig. 2e, f); whereas, knockdown of CTSL expression in GC cells led to the reduced migratory capacity of HUVECs (Fig. 2g, h). Furthermore, a wound-healing assay confirmed that knockdown of CTSL in GC cells markedly increased HUVECs migration (Fig. 2i–m, Supplementary Fig. 3A); whereas, overexpression had the opposite effect (Supplementary Fig. 3B and Fig. 2n). These results indicate that CTSL could promote the tubular formation and migration of HUVEC cells in vitro.

### CTSL promotes angiogenesis and tumor growth in vivo

**Fig. 3:**
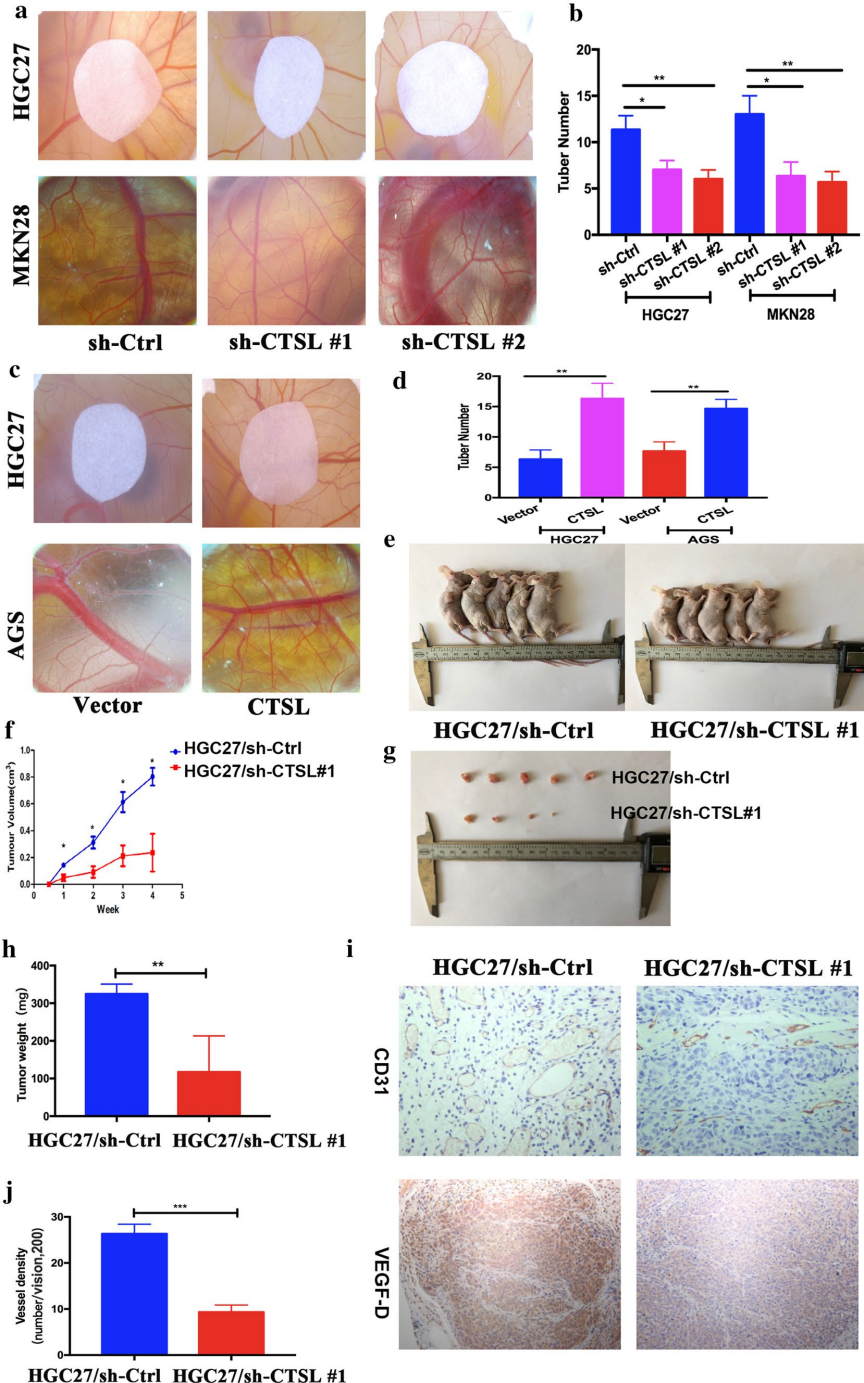
CTSL promoted angiogenesis and tumor growth in vivo. **a**, **b** The CAM assay was performed to investigate the effect of CM from GC cells after transfection with CTSL shRNA plasmids (HGC27 and MKN28) on HUVEC tubule formation in vivo. **c**, **d** The CAM assay was performed to investigate the effect of CM from GC cells after transfection with CTSL-expressing plasmids (HGC27 and AGS) on HUVEC tubule formation in vivo. **e**, **f** Mice were injected with HGC27 cells transfected with CTSL shRNA plasmids (sh-CTSL #1) or sh-Ctrl, and tumor volumes were monitored weekly. **g**, **h** Average tumor weights from nude mice injected with HGC27/sh-CTSL cells or HGC27/sh-Ctrl cells. **i** Representative images of CD31 (PECAM1) and VEGF-D immunostaining of tumor samples from different tumor model mice groups. **j** Analysis of VD in different tumor model mice groups showed that VD was significantly higher in tumors from HGC27/sh-Ctrl compared with tumors from HGC27/sh-CTSL

To determine the effect of CTSL on angiogenesis in vivo, we performed a chick embryo CAM assay, which has emerged as a standard in in vivo angiogenesis assay [32]. Treatment with CM from the CTSL-knockdown GC cells significantly reduced the vessel number compared with their respective control group (Fig. 3a, b); whereas, CM from CTSL-overexpressing GC cells induced the formation of more vessels than that from their respective control group (Fig. 3c, d). In addition, we further identified the tumor-promoting effect of CTSL in vivo. We subcutaneously transplanted HGC27/sh-CTSL and HGC27/sh-Ctrl cells into nude mice. As shown in Fig. 3e–h, the tumors generated by HGC27/sh-CTSL cells exhibited smaller volume and less weight than those generated by HGC27/sh-Ctrl cells. Moreover, we further detected VD in subcutaneous tumors derived from HGC27/sh-CTSL and HGC27/sh-Ctrl cells. Anti-CD31 immunostaining revealed that vascellum in tumors from HGC27/sh-Ctrl cells was more developed; however, less vascellum formed in tumors from HGC27/sh-CTSL cells (Fig. 3i). Consistently, the assessment of averaged VD per field in each group indicated a threefold higher level in tumors from HGC27/sh-Ctrl cells when compared with tumors from HGC27/sh-CTSL cells (Fig. 3j). Thus, these findings suggest that CTSL in GC cells enhances tumor progression by promoting the angiogenesis of GC.

### CTSL promotes angiogenesis by transcriptionally activating of VEGF-D via p110 Cux1 in GC cells

**Fig. 4:**
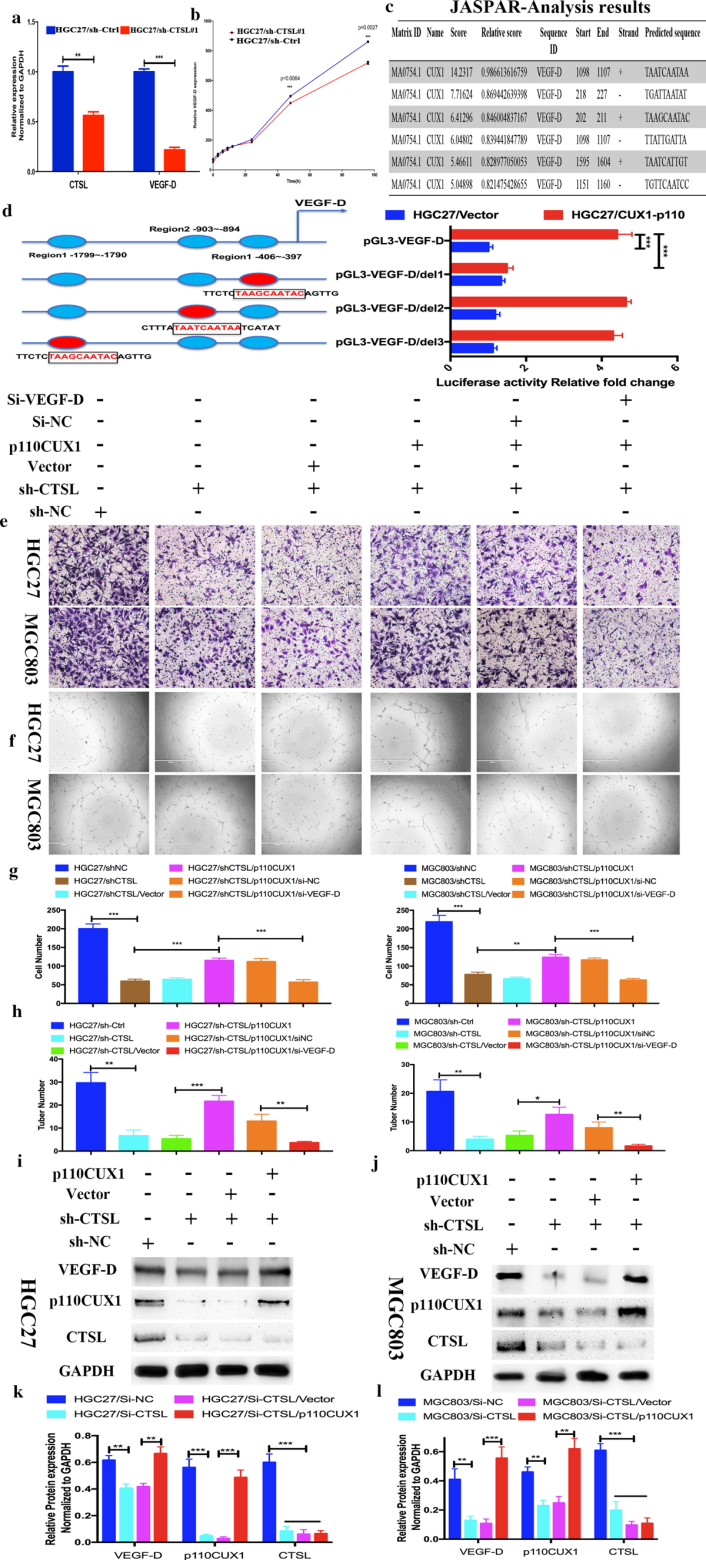
CTSL promoted the transcriptional activation of VEGF-D in GC cells by the processing of CUX1. **a** qRT-PCR assays showed that the mRNA levels of VEGF-D were obviously downregulated in the HGC27/sh-CTSL group compared with the HGC27/sh-Ctrl group. **b** ELISA demonstrated that the secretion of VEGF-D was decreased in the HGC27/sh-CTSL group compared with the HGC27/sh-Ctrl group. **c** The predicted potential binding sites between CDP/CUX and VEGF-D. **d** Deletion analysis at positions − 406 to − 397 bp (designated site 1), − 903 to − 894 bp (designated site 2), and − 1799 to − 1790 bp (designated site 3) identified three CDP/CUX1-responsive regions in the human VEGF-D promoter (left). pGL3-VEGF-D, pGL3-VEGF-D-del 1 (deletion at positions − 1799 to − 1790), pGL3-VEGF-D-del 2 (deletion at positions − 903 to − 894 bp), pGL3-VEGF-D-del 3 (deletion at positions − 406 to − 397 bp) constructs, along with the pRL-TK plasmid, were transfected into HGC27-p110CUX1 cells and parental HGC27 cells, and then the relative luciferase activity was measured (right). **e**, **g** In vitro Transwell migration assay to investigate the effect of CM from GC cells (MGC803 and HGC27) after they were transfected with sh-CTSL, p110CUX1-expressing vector, and si-VEGF-D on the metastatic abilities of HUVEC. Data are presented as the mean ± SD; *n* = 3 independent experiments. **f**, **h** In vitro tubular formation to investigate the effect of CM from GC cells (MGC803 and HGC27) after they were transfected with sh-CTSL, p110CUX1-expressing vector, and si-VEGF-D on HUVEC tubule formation. Data are presented as the mean ± SD; *n* = 3 independent experiments. **i–l** The expression levels of CTSL, p110CUX1, and VEGF-D proteins in MGC803 and HGC27 cells transfected with p110CUX1-expressing vector or CTSL shRNA were analyzed by Western blot. Data are presented as the mean ± SD; *n* = 3 independent experiments

The angiogenesis of a tumor is regulated by the coordination of the angiogenic and anti-angiogenic factors secreted by tumor cells or stromal cells in tumor microenvironment. To identify the factors from GC cells involved in the angiogenesis of tumors, we performed a human angiogenesis antibody array on CM from HGC27/sh-CTSL and HGC27/sh-Ctrl cells. The result showed a remarkable decrease in VEGF-D in CM from HGC27/sh-CTSL cells (Supplementary Fig. 4A and 4B). We, therefore, quantified the expression level of VEGF-D in HGC27/sh-CTSL cells and HGC27/sh-Ctrl cells by qRT-PCR, and we found a fivefold reduction of VEGF-D mRNA in HGC27/sh-CTSL cells compared with HGC27/sh-Ctrl cells (Fig. 4a). In addition, we collected the CM from HGC27/sh-CTSL cells and HGC27/sh-Ctrl cells and detected the protein expression of VEGF-D in CM by enzyme-linked immunosorbent assay (ELISA). The result also showed that silencing CTSL led to a significant decrease in VEGF-D secretion by GC cells (Fig. 4b).

CCAAT-displacement protein/cut homeobox (CDP/Cux) belongs to a family of transcription factors and has four evolutionarily conserved DNA binding domains: the Cut homeodomain (HD) and the three Cut repeats (CR1, CR2, and CR3) [11]. The full-length protein, named p200 Cux1, is abundant but only transiently binds DNA [23]. P110 Cux1, proteolytic processing of p200 Cux1 by CTSL, stably interacts with DNA, and functions as a transcriptional repressor or activator depending on promoter context [26, 29]. To identify whether upregulating CTSL expression in GC cells could increase CDP/Cux degradation to p110 Cux1, we performed Western blot to detect a change in the protein level of CDP/Cux. We found that overexpression of CTSL in GC cells (MGC803 and HGC27) promoted the cleavage of CDP/Cux and significantly increased the expression of p110 Cux1 isoform (Supplementary Fig. 4C and 4D). To further determine whether p110 Cux1 could directly bind the VEGF-D promoter and transcriptionally activate its expression in GC cells, we next analyzed the promoter region of VEGF-D, which ranged from − 2000 to + 100 bp with respect to the transcription start site (TSS), using the JASPAR database (Fig. 4c). We identified three putative p110 Cux1 binding sites (score > 5) at site A (− 1799 to − 1790), site B (− 903 to − 894), and site C (− 406 to − 397). Dual-luciferase reporter assay showed that p110 Cux1 significantly increased the relative luciferase activity compared with the control group by cotransfecting p110 Cux1 and the full-length promoter of VEGF-D, the promoter 1 vector (deletion at site B: − 903 bp to − 894 bp), or the promoter 2# vector (deletion site C: − 406 to − 397). Deletion at site A (− 1799 bp to − 1790 bp), however, significantly decreased p110 Cux1-induced VEGF-D promoter activity (Fig. 4d), which demonstrated that p110 Cux1 could bind to the promoter of VEGF-D at − 1799 to − 1790 bp and then could activate VEGF-D transcription.

To further evaluate whether CTSL promotes the angiogenic ability of HUVECs through the CDP/Cux/VEGF-D pathway, we first established an in vitro co-culture system. We indirectly co-cultured HUVECs with GC cells (HGC27 and MGC803) after transfection with sh-CTSL, p110 Cux1-expressing vector, or Si-VEGF-D. Next, we performed tubule formation and Transwell assays, and the results revealed that upregulation of p110 Cux1 in GC cells promoted the tubule formation and migratory ability of HUVECs, which could be reversed by co-transfection with Si-VEGF-D (Fig. 4e–h). To ascertain whether CTSL promotes the transcription of VEGF-D by increasing the expression of p110 Cux1, we next performed Western blot and the results showed that the overexpression of p110 Cux1 can rescue the sh-CTSL-mediated suppression of VEGF-D in HGC27 and MGC803 GC cells (Fig. 4i–l). In general, we found that CTSL enhances the cleavage of CDP/Cux to p110 Cux1, which promotes the transcriptional activation of VEGF-D to subsequently induce angiogenesis of GC.

### CTSL expression is positively correlated with VEGF-D in GC tissues

**Fig. 5:**
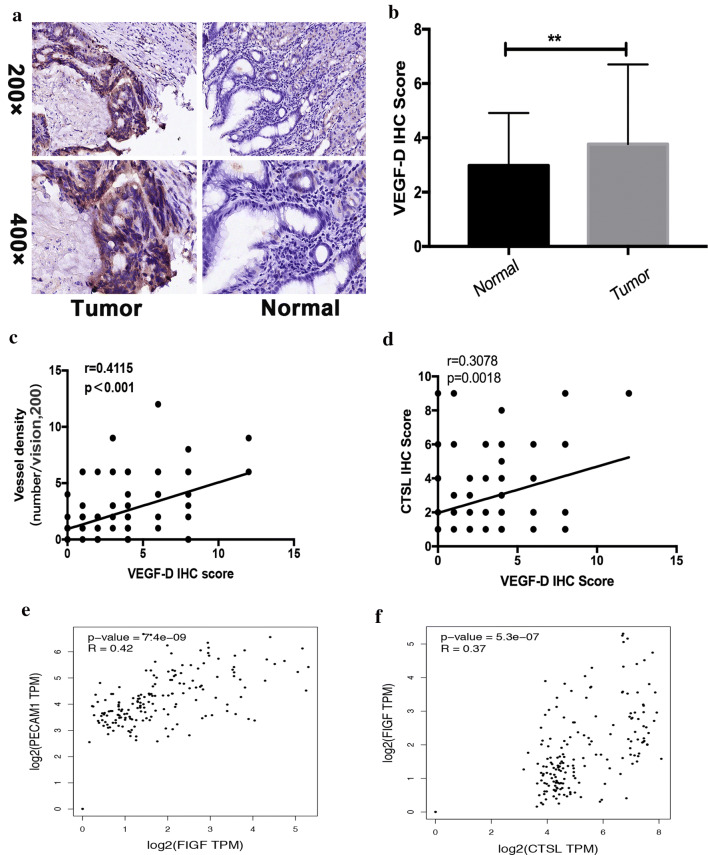
CTSL was positively correlated with VEGF-D in GC tissues. **a** Representative IHC staining with VEGF-D antibody in GC and paired adjacent nontumor tissues. Magnification: × 200 and × 400. **b** Analysis of VD in 134 pairs of GC patients (GC cohort 1) showed that VEGF-D was significantly higher in GC tissues compared with adjacent nontumor tissues. **c** Correlation analysis of VEGF-D and VD showed that VEGF-D was positively correlated with VD in GC. **d** Correlation analysis of VEGF-D and CTSL showed that VEGF-D was positively correlated with CTSL in GC. **e** Validation of the positive correlation between VEGF-D and VD in GEPIA. **f** Validation of the positive correlation between VEGF-D and CTSL in GEPIA

We further examined the correlation between the expression level of CTSL and VEGF-D in human GC. We performed IHC analysis to check VEGF-D expression in 157 GC patients (GC cohort 2), and the results revealed that VEGF-D expression was significantly upregulated in GC tissues compared with adjacent non-tumor tissues (Fig. 5a, b). Moreover, we found that the expression level of VEGF-D in GC was positively correlated with VD and the expression of nuclear CTSL (Fig. 5c, d). Similarly, bioinformatic analysis of the correlation of VEGF-D and VD revealed that VEGF-D was positively correlated with VD in GC (Fig. 5e). In addition, we analyzed the correlation between CTSL and VEGF-D in GC using TCGA data. As shown in Fig. 5f, CTSL expression was positively correlated with the expression of VEGF-D.

## Discussion

CTSL belongs to the papain-like family of cysteine proteases and is essential in the terminal degradation of proteins [33]. CTSL localizes primarily to the lysosomes and is also a constituent of the nuclear protein fraction. Although previous studies have shown that CTSL is upregulated in GC and could be identified as a marker of enhanced invasiveness of GC [10, 15, 30], the function of nuclear CTSL in GC angiogenesis has not been determined. In this study, we found that nuclear CTSL is significantly upregulated in GC cancer and predicts poor prognosis of GC patients. Nuclear CTSL significantly promotes the angiogenic potential of HUVECs cells in vitro and in vivo by promoting CDP/Cux degradation to the p110 Cux1 isoform, thus increasing the transcription of VEGF-D. In this study, we first showed that CTSL plays a facilitative role in regulating tumor angiogenesis of GC and that it could serve as an independent prognostic indicator and therapeutic target for GC.

Functions of cysteine cathepsins depend on their subcellular localization. For example, cysteine cathepsins are involved in cell death and inflammation in the cytoplasm, and they also regulate cell cycle in the nucleus and exert degradative roles in the extracellular environment [18]. CTSL is a ubiquitously expressed cysteine protease that localizes primarily to the lysosome. The role of CTSL originally was assumed to be terminal degradation of proteins in the lysosomal compartment. CTSL expression and subcellular localization, however, do not appear to be uniform. Haugen et al. identified CTSL as a constituent of the nuclear protein fraction [12]. In this study, we found that nuclear CTSL promoted angiogenesis by enhancing the cleavage of CDP/Cux to p110 Cux1 in GC. Proteolytic processing is an important post-translational mechanism that modulates downstream signaling. CDP/Cux can be proteolytically processed by CTSL, thereby generating the p110 Cux1 isoform that contains three DNA-binding domains, CR2, CR3, and HD. CDP/Cux can bind only transiently to DNA, and the consequence of proteolytic processing is to activate the ability of CDP/Cux to interact stably with DNA. The p110 Cux1 processed isoform binds stably to DNA, and therefore, it can function like a classical transcription factor [23]. Thus, we provided a novel insight into the proteolytic activity of nuclear CTSL in GC cells. The mechanism by which nuclear CTSL regulates the progression of GC requires further study.

VEGF-D is a secreted glycoprotein that promotes growth of blood vessels (angiogenesis) and lymphatic vessels (lymphangiogenesis). VEGF-D enhances tumor growth and metastatic spread, which correlates with poor patient outcome and resistance to anti-angiogenic drugs. VEGF-D activates VEGF receptors (VEGFR-2 and VEGFR-3) on the endothelium to promote the growth and remodeling of blood or lymphatic vessels [28]. VEGF-D has been reported to be an independent prognostic marker, which aids in the identification of patients with poor prognosis after curative resection of gastric adenocarcinomas. Consistent with the previous study, we found that VEGF-D promoted the angiogenesis in GC. Although the molecular mechanisms that regulate expression of VEGF-D have been gradually interpreted, they have been not fully understood in GC. In this study, we identified that p110 Cux, an isoform of CDP/Cux1, was a transcription factor that specifically promoted the transcription of VEGF-D. Thus, our data uncovered a novel mechanism underlying VEGF-D regulation in GC.

## Conclusion

Our findings demonstrated that nuclear CTSL was significantly upregulated in GC tissues and enhanced the cleavage of CDP/Cux to p110 Cux1 isoform by its proteolytic activity in GC cells. Moreover, we identified for the first time that p110 Cux1 could bind to the promoter of VEGF-D and subsequently activated VEGF-D transcription, which led to GC angiogenesis.

## Electronic supplementary material

Below is the link to the electronic supplementary material.

Supplementary Figure S1. The expression of CTSL in pan-cancer. (TIF 626 kb)

Supplementary Figure S2. The expression profiles in GC cell lines and diagram of the in vitro coculture system. A and B: Western Blot analysis of CTSL expression levels in GC cell lines (SUN-1, HGC27, AGS, MKN45, MGC803, MKN28, and NCI-N87) and a normal gastric epithelium cell line (GES-1). Data are presented as the mean ± SD; n = 3 independent experiments. C: In vitro coculture system. (TIF 202 kb)

Supplementary Figure S3. CTSL promoted the migration of HUVEC cells. A: Scratch wound-healing motility assays were performed to observe the changes in the migration of HUVEC after stimulated with CM from GC cells after transfection with CTSL shRNA plasmids (MKN28). B: Scratch wound-healing motility assays were performed to observe the changes in the migration of HUVEC after stimulation with CM from GC cells after transfection with CTSL-expressing plasmids (AGS). (TIF 989 kb)

Supplementary Figure S4. CTSL degraded the CDP/CUX protein and bioinformatic analysis predicted the potential binding sites between CDP/CUX and VEGF-D. A: Human Angiogenesis Antibody Array identified differentially expressed angiogenesis factors in the CM of HGC27/sh-Ctrl and HGC27/sh-CTSL, as shown by the blots. B: Heatmap of human angiogenesis antibody array. C and D: The CDP/CUX1 protein levels in MGC803 and HGC27cells were separately detected after transfection of CTSL expression and shRNA plasmids. Data are presented as the mean ± SD; n = 3 independent experiments. (TIF 1064 kb)

## Data Availability

All data and details can be obtained by contacting the corresponding author.

## Abbreviations

CTSL: Cathepsin L
GC: Gastric cancer
VEGF-D: Vascular endothelial growth factors D
CDP/Cux1: CCAAT displacement protein/cut like homeobox 1
FBS: Fetal bovine serum
