# Supplementary figures and images for: Cathepsin L promotes angiogenesis by regulating the CDP/Cux/VEGF-D pathway in human gastric cancer
Source: Gastric Cancer. 2020 May 9;23(6):974–87. doi: 10.1007/s10120-020-01080-6 (PMC7567730; doi:10.1007/s10120-020-01080-6)

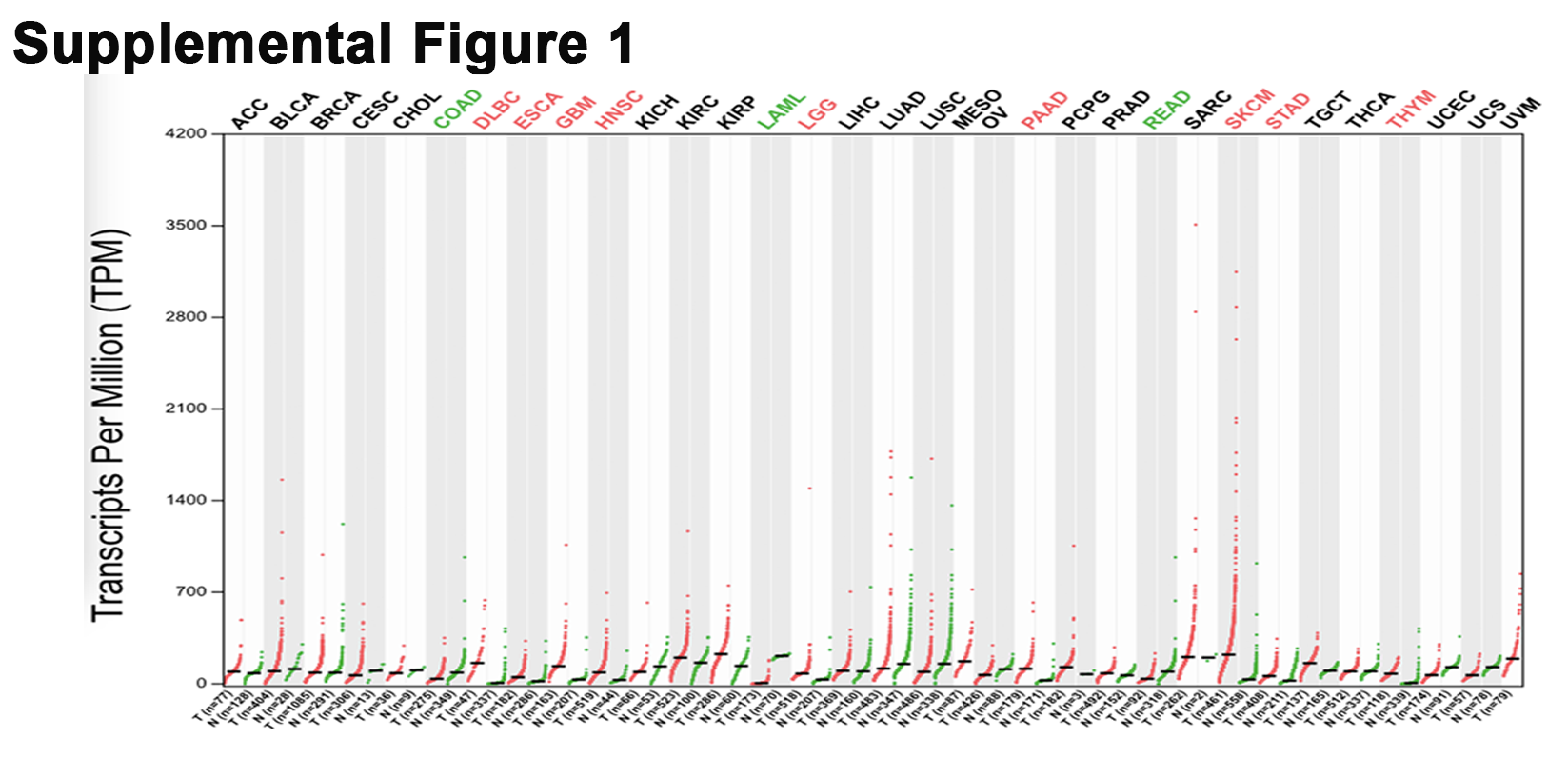

Supplement: Supplementary file 1 — Supplementary Figure S1. The expression of CTSL in pan-cancer. (TIF 626 kb) [file 10120_2020_1080_MOESM1_ESM.tif]

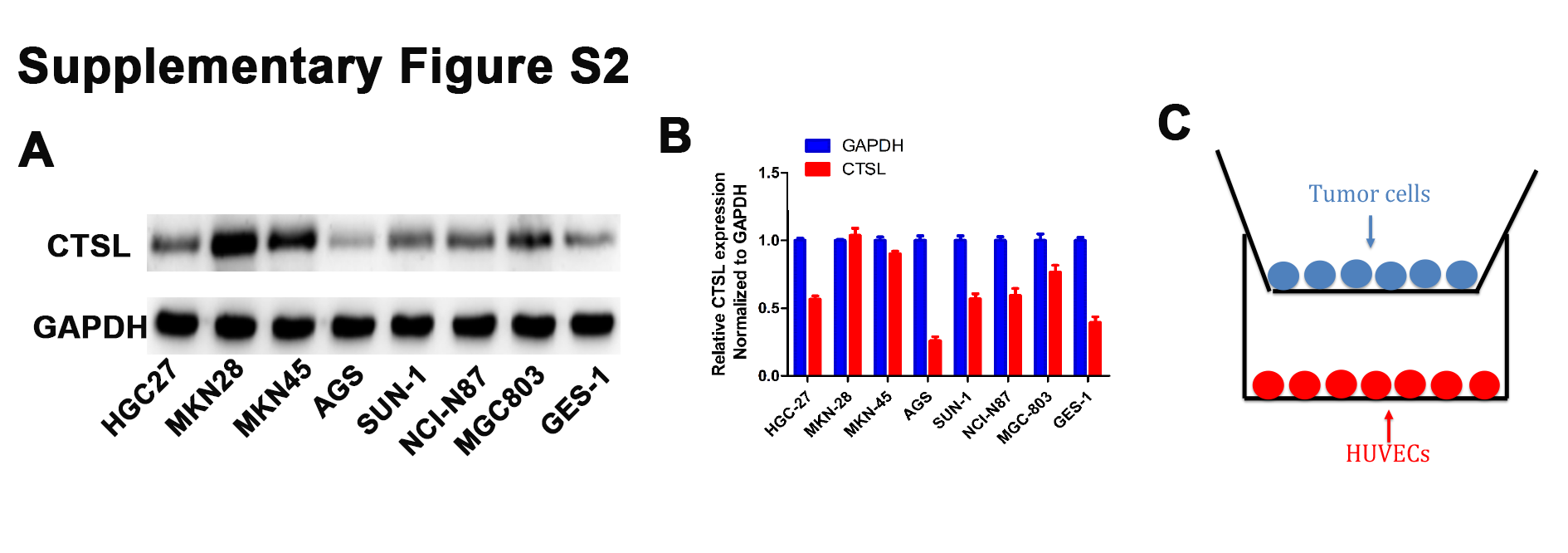

Supplement: Supplementary file 2 — Supplementary Figure S2. The expression profiles in GC cell lines and diagram of the in vitro coculture system. A and B: Western Blot analysis of CTSL expression levels in GC cell lines (SUN-1, HGC27, AGS, MKN45, MGC803, MKN28, and NCI-N87) and a normal gastric epithelium cell line (GES-1). Data are presented as the mean ± SD; n = 3 independent experiments. C: In vitro coculture system. (TIF 202 kb) [file 10120_2020_1080_MOESM2_ESM.tif]

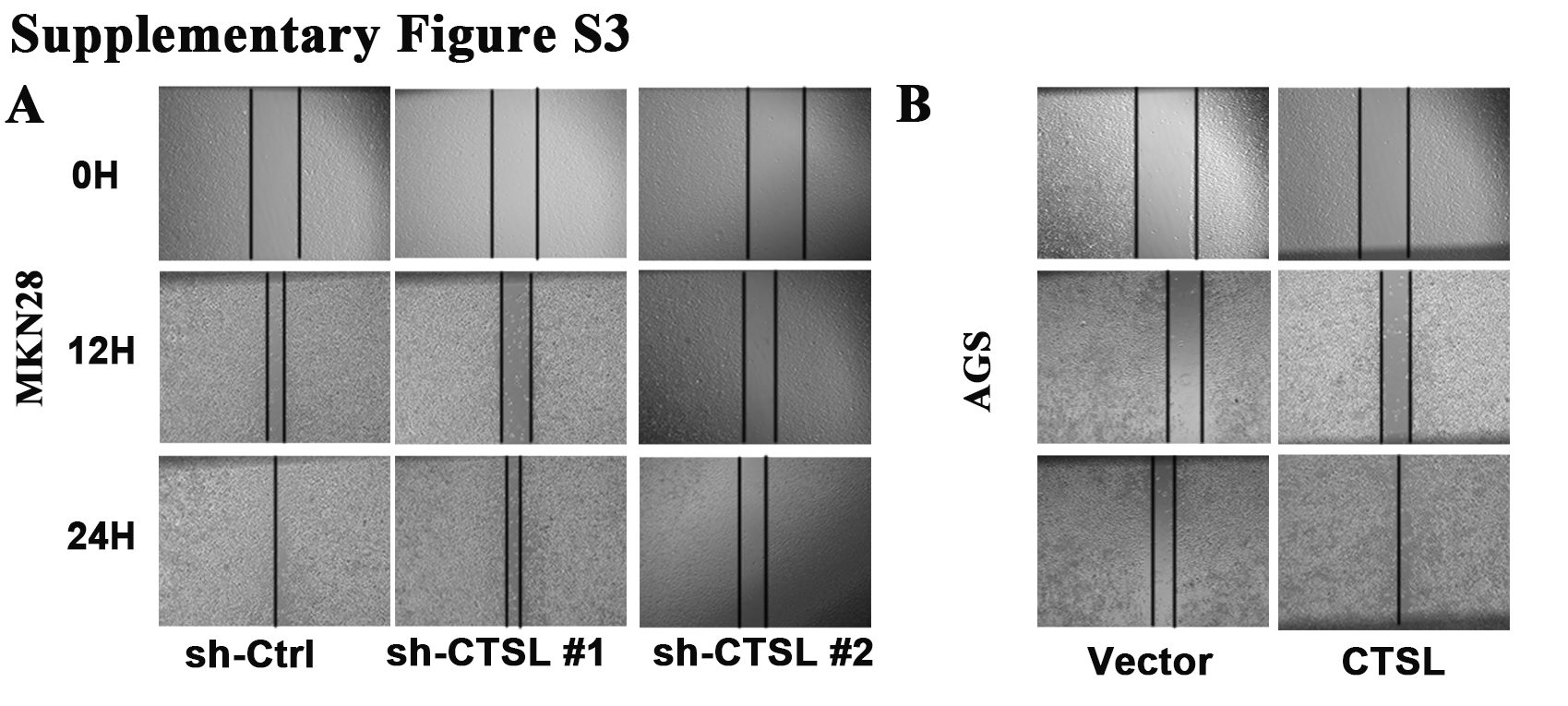

Supplement: Supplementary file 3 — Supplementary Figure S3. CTSL promoted the migration of HUVEC cells. A: Scratch wound-healing motility assays were performed to observe the changes in the migration of HUVEC after stimulated with CM from GC cells after transfection with CTSL shRNA plasmids (MKN28). B: Scratch wound-healing motility assays were performed to observe the changes in the migration of HUVEC after stimulation with CM from GC cells after transfection with CTSL-expressing plasmids (AGS). (TIF 989 kb) [file 10120_2020_1080_MOESM3_ESM.tif]

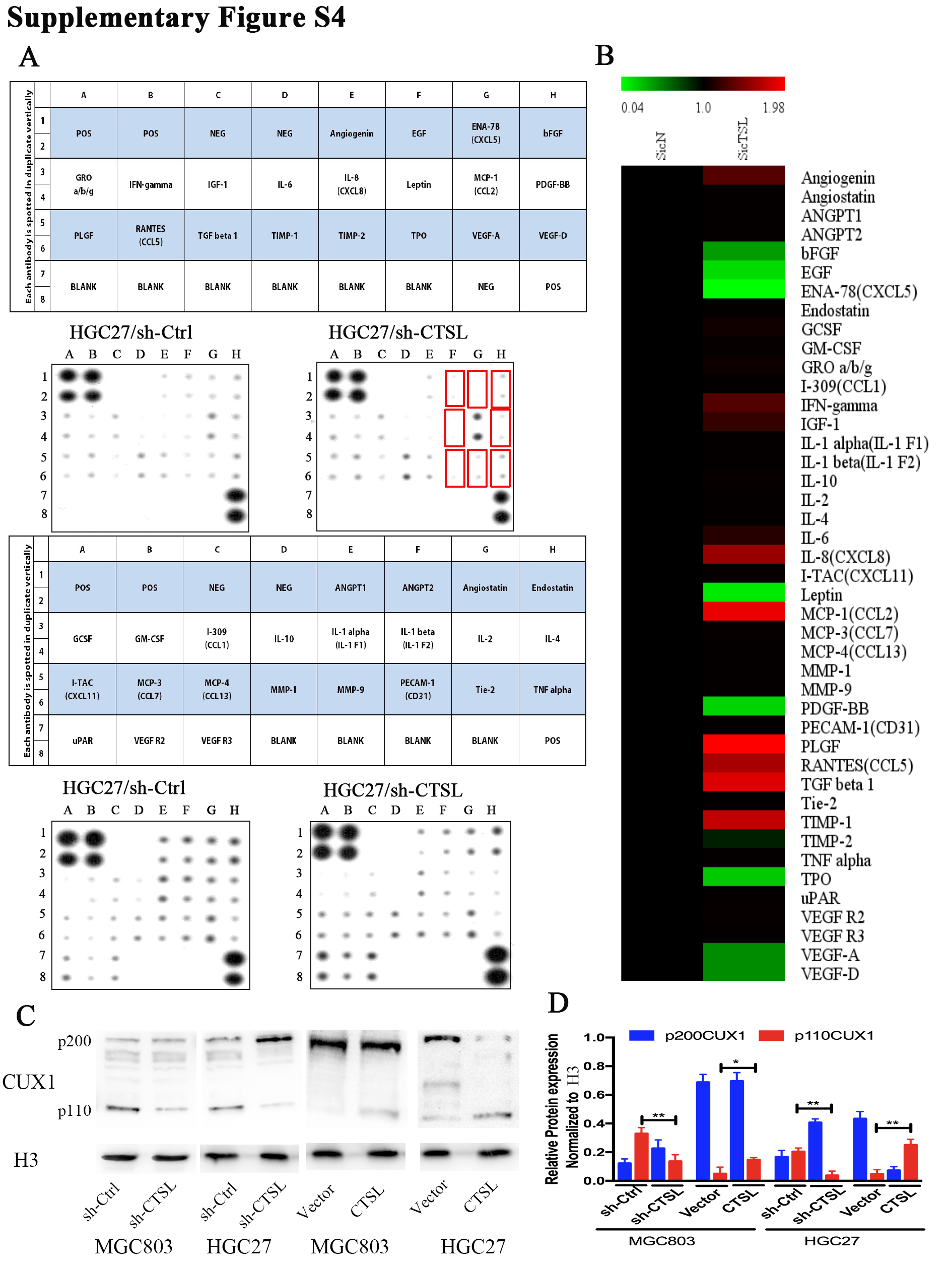

Supplement: Supplementary file 4 — Supplementary Figure S4. CTSL degraded the CDP/CUX protein and bioinformatic analysis predicted the potential binding sites between CDP/CUX and VEGF-D. A: Human Angiogenesis Antibody Array identified differentially expressed angiogenesis factors in the CM of HGC27/sh-Ctrl and HGC27/sh-CTSL, as shown by the blots. B: Heatmap of human angiogenesis antibody array. C and D: The CDP/CUX1 protein levels in MGC803 and HGC27cells were separately detected after transfection of CTSL expression and shRNA plasmids. Data are presented as the mean ± SD; n = 3 independent experiments. (TIF 1064 kb) [file 10120_2020_1080_MOESM4_ESM.tif]
